# Acute central retinal artery occlusion presenting as CREST syndrome: a case report

**DOI:** 10.1186/1757-1626-2-9

**Published:** 2009-01-05

**Authors:** Muhammad SA Raja, Tarnya Marshall, Ben JL Burton

**Affiliations:** 1 James Paget University Hospital NHS Foundation Trust, Lowestoft Road, Gorleston, Great Yarmouth NR31 6LA, Norfolk, UK; 2 Norfolk and Norwich University Hospital NHS Trust, Colney Lane, Norwich NR4 7UY, Norfolk, UK

## Abstract

**Background:**

A 75 year old lady presented with acute central retinal artery occlusion and contralateral cotton wool spots.

**Case presentation:**

General physical examination and investigations led to a diagnosis of CREST syndrome; however, association of central retinal artery occlusion with CREST syndrome is not well known. While diabetes, systemic hypertension, carotid atherosclerosis and cardiac pathology are common causes of CRAO it is always important to rule out giant cell arteritis.

**Conclusion:**

This case highlights that inflammatory causes of central retinal artery occlusion other than giant cell arteritis should also be considered as a possibility to spare unnecessary use of excessive systemic corticosteroids.

## Background

We present an unusual case of acute central retinal artery occlusion and cotton wool spots as the presenting feature in a patient subsequently diagnosed with systemic sclerosis, in particular with features of CREST syndrome.

## Case presentation

A 75-year old Caucasian lady presented with acute onset of painless visual loss in her left eye, which she noted on waking up that morning. She denied any associated headaches, jaw claudication or any clinical symptoms suggestive of temporal arteritis. Past ophthalmic history was not significant apart from one episode of transient visual loss in the left eye of about 5 minutes duration with zigzag lines three months earlier. This was accompanied by spontaneously resolving short-duration headache. The general practitioner diagnosed an episode of migraine with no further investigations or recurrence. She had treated essential hypertension and suffered especially in cold weather with Raynaud's phenomenon for 20 years; this had worsened in severity in the last few months.

**Figure 1:**
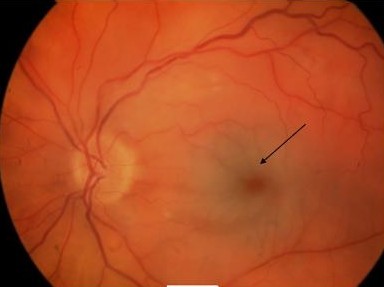
**Left eye showing cherry-red spot with retinal pallor typical of central retinal artery occlusion (arrow)**.

**Figure 2:**
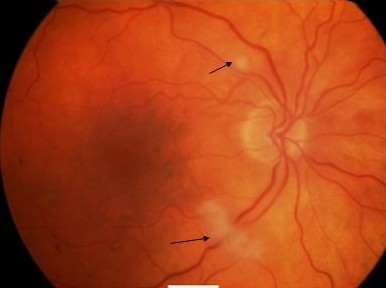
**Right eye showing cotton-wool spots noted along superotemporal and inferotemporal arterioles (arrows)**.

**Figure 3:**
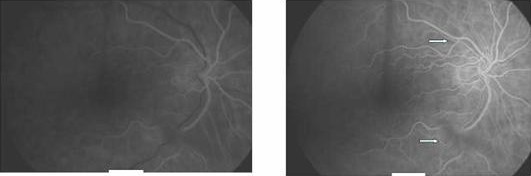
**FFA of the right eye showing good choroidal filling in early phase (left) and blocked hyperfluorescence due to cotton-wool spots (arrows)**.

**Figure 4:**
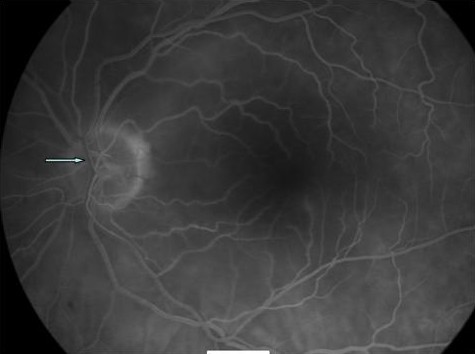
**FFA in the late phase of the left eye delayed venous filling (arrow) but no significant leakage or staining**.

On examination visual acuity of 20/20 in the right and CF (counting fingers at 0.5 m) was noted in the left eye with a left relative afferent pupillary defect. Anterior segment examination was unremarkable with normal intraocular pressures. Dilated fundal examination showed a cherry-red spot in the left foveal area. No visible arteriolar embolus was noted (Fig 1). Apart from presence of two cotton wool spots in the right eye the fundal examination was unremarkable (Fig 2). Superficial temporal arteries were palpable, non-tender and not thickened or nodular with good flow. Cardiac examination & carotid artery auscultation was unremarkable. A clinical diagnosis of left acute central retinal artery occlusion with cotton wool spots in the right eye led to a suspected diagnosis of temporal arteritis. Urgent blood investigations were requested but the ESR, CRP, platelets counts and liver function tests were within normal limits. Fundus fluorescein angiography was carried out on the third day showing no evidence of choroidal ischaemia (Fig 3 and 4). She was initially started on 80 mg of oral prednisolone daily but with availability of all the blood tests, the angiogram features and lack of any clinical symptoms, the steroids were stopped after 3 days as temporal arteritis was deemed unlikely. She was commenced on 150 mg of aspirin. A closer examination of her hands showed tethered skin with spindly fingers. She was duly referred for a rheumatology opinion to rule out any vasculitic aetiology linking her hands, Raynaud's & ophthalmological features.

Further investigations revealed a positive ANA's at 8.1 U (0–1.0 reference range) with a positive anti-centromere antibody and a negative anti-Scl-70 and anti-ds DNA antibody. X-rays of the hands did not reveal any significant calcinosis or erosions. Barium swallow was unremarkable but echocardiography showed mild concentric hypertrophy of ventricular walls consistent with long-standing systemic hypertension.

Mild non-flow limiting plaque was also detected on coronary angiography. Her pulmonary hypertension was attributed to her cardiac status with atrial fibrillation and systemic hypertension rather than connective tissue disorder related. Carotid duplex scans revealed minor calcific plaques with no significant stenosis in either carotid arterial system. She remains under joint rheumatology and ophthalmology review on immunosuppression with methotrexate 10 mg per week, prednisolone 7.5 mg daily and anti-platelet therapy with no further visual symptoms after 12 months of follow up.

## Discussion

Various authors [1-5] have noted retinal venous occlusions and choroidal circulation compromise as possible associations with systemic sclerosis spectrum but central retinal arterial occlusion, to our knowledge, has not been reported as the presenting feature of CREST syndrome.

CREST syndrome can be quite variable and may or may not show all the features in a single patient at one time point. Our case highlights the importance of thorough clinical examination including review of systems and appropriate referral as the appearance of her hands, telangiectasias & presence of Raynaud's phenomenon lead to well-directed investigations and subsequent clinical diagnosis. While temporal arteritis was a reasonable diagnosis to entertain initially, we stopped the oral steroids immediately with unremarkable blood and angiogram results. The patient was thus saved a significant systemic corticosteroid load with avoidance of possible adverse effects including scleroderma renal crisis which may be seen in scleroderma patients treated with high dose steroids.

The presence of cotton wool spots in her right eye strongly suggested an inflammatory cause for CRAO and although microvasculopathy is a more consistent feature of the systemic sclerosis spectrum, macrovascular disease has been reported. It should be noted that in general CRAO is rarely caused by inflammation with atherosclerotic disease being the usual aetiology [4-7]. In our patient no significant carotid artery stenosis was noted on duplex scans but small plaques were visualized and retinal micro-embolism can originate from the tiny plaques causing retinal artery occlusion. It is important not to discard the absence of significant stenosis to rule out implicating carotid disease for arterial occlusion. Perfusion deficit caused by systemic nocturnal hypotension especially with patients taking nocturnal anti-hypertensive medication is another unlikely possibility. Cardiac emboli can be a cause of CRAO but our patient had mild cardiac disease and no features of cardiac valve pathology or evidence of calcific emboli.

In a review [5] listing 52 potential associations &/or causes of retinal arterial obstruction of varying pathophysiology, systemic sclerosis did not find a mention in the collagen vascular/other vasculitides group. Importantly, the authors emphasize commoner associations as diabetes, systemic hypertension, carotid atherosclerosis and cardiac sources of thromboembolism. Giant cell arteritis remains an important cause and is a potentially blinding condition but needs long-term systemic corticosteroid therapy, which has potentially serious side effects.

## Conclusion

Ophthalmologists should be aware of this unusual association between central retinal artery occlusion and CREST syndrome. While the prognosis for central retinal artery occlusion is extremely guarded, systemic workup to rule out life threatening co-morbid conditions remains the cornerstone of management.

## Abbreviations

CREST: (Calcinosis, Raynaud's phenomenon, Oesophageal dysmotility, Sclerodactyly, Telangiectasias); CRAO: (Central retinal artery occlusion); ESR: Erythrocyte sedimentation rate; CRP: C Reactive Protein; ANA: (Anti-Nuclear antibodies); Anti-ds: (Anti-double strand).

## Competing interests

The authors declare that they have no competing interests.

## Authors' contributions

MR analyzed and reviewed the literature and drafted the case report. TM and BJB provided valuable input to the manuscript. All authors read and approved the final manuscript.

## Consent

Written informed consent was obtained from the patient for the publication of this case report and accompanying images. A copy of the written consent is available for review by the Editor-in-Chief of this journal.
